# Biophotons and Emergence of Quantum Coherence—A Diffusion Entropy Analysis

**DOI:** 10.3390/e23050554

**Published:** 2021-04-29

**Authors:** Maurizio Benfatto, Elisabetta Pace, Catalina Curceanu, Alessandro Scordo, Alberto Clozza, Ivan Davoli, Massimiliano Lucci, Roberto Francini, Fabio De Matteis, Maurizio Grandi, Rohisha Tuladhar, Paolo Grigolini

**Affiliations:** 1 Laboratori Nazionali di Frascati, Istituto Nazionale di Fisica Nucleare, Via E. Fermi 40, 00044 Frascati, Italy; elisabetta.pace@lnf.infn.it (E.P.); Catalina.Curceanu@lnf.infn.it (C.C.); alessandro.scordo@lnf.infn.it (A.S.); alberto.clozza@lnf.infn.it (A.C.); 2 Dipartimento di Fisica, Università di Roma “Tor Vergata”, Via della Ricerca Scientifica, 00133 Roma, Italy; Ivan.Davoli@roma2.infn.it (I.D.); massimiliano.lucci@roma2.infn.it (M.L.); 3 Dipartimento di Ingegneria Industriale, Università di Roma “Tor Vergata”, Via del Politecnico, 00133 Roma, Italy; roberto.francini@roma2.infn.it (R.F.); dematteis@roma2.infn.it (F.D.M.); 4 Istituto La Torre, Via M. Ponzio 10, 10141 Torino, Italy; mauriziograndi@mauriziograndi.it; 5 Department of Biology, University of Texas at San Antonio, San Antonio, TX 78249, USA; raisha.t@gmail.com; 6 Center for Nonlinear Science, University of North Texas, Denton, TX 76203-5017, USA

**Keywords:** biophotons, diffusion entropy analysis, complexity, cognition

## Abstract

We study the emission of photons from germinating seeds using an experimental technique designed to detect light of extremely small intensity. We analyze the dark count signal without germinating seeds as well as the photon emission during the germination process. The technique of analysis adopted here, called diffusion entropy analysis (DEA) and originally designed to measure the temporal complexity of astrophysical, sociological and physiological processes, rests on Kolmogorov complexity. The updated version of DEA used in this paper is designed to determine if the signal complexity is generated either by non-ergodic crucial events with a non-stationary correlation function or by the infinite memory of a stationary but non-integrable correlation function or by a mixture of both processes. We find that dark count yields the ordinary scaling, thereby showing that no complexity of either kinds may occur without any seeds in the chamber. In the presence of seeds in the chamber anomalous scaling emerges, reminiscent of that found in neuro-physiological processes. However, this is a mixture of both processes and with the progress of germination the non-ergodic component tends to vanish and complexity becomes dominated by the stationary infinite memory. We illustrate some conjectures ranging from stress induced annihilation of crucial events to the emergence of quantum coherence.

## 1. Introduction

### 1.1. Introduction to Biophotons

Nearly a hundred years ago the Russian biologist A. Gurwitsch put forth the hypothesis that a weak ultraviolet (UV) radiation comes out from the living tissues, and it is the responsible for the regulation of cell growth [1,2]. He called this radiation “mitogenetic radiation”. However, this interestin result was forgotten by the scientific community and mitogenetic radiation has been considered as an artifact for many years. With the improvement of the methods to detect weak level of radiation and the increase in understanding of quantum optics, there has been a renewed interest in this phenomenon. Colli and Facchini in the 1950s [3,4] and Popp [5] in the 1980s did extensive work in this field to understand in details the origin and the meaning of such ultra-weak emissions, hereby called bio-photons. Bio-photons are an endogenous production of photon in and from cells and organisms, and this emission is characteristic of alive organisms. This emission is entirely different from the normal bioluminescence observed in some simple as well complex organisms, it is at least 1000 times weaker, and it is present in all living organism, from plants to human beings. It is also different from the thermal radiation because its intensity is many orders of magnitude higher than the one calculated by Planck’s law in the visible energy range at room temperature.

The main characteristics of biophotons measured up to now are the following [5,6]: the total intensity of the emission is quite low and goes from several to some hundred photons/sec per cm^2^ surface of the living system. The spectral intensity seems to be quite flat for wavelengths ranging between 200 and 800 nm. After any type of stress (chemical agents, excitation by white or monochromatic light, temperature…) the emission increases of almost a factor ten and relaxes to the normal values quite slowly, following hyperbolic functions rather than an exponential law. Finally, the photocount statistics that account for the probability of having N photons within some time interval seems to follow a Poisson distribution. The biophoton emission is also very sensitive to the biological characteristics of the system under examination, and for this reason, this types of measures has been successfully applied to medical diagnostics, agriculture and ecology [7,8,9].

Despite the wealth of experimental results, the questions of what biophotons are, how they are generated and how they are involved with life are, in our opinion, still open.

Actually, there are two hypotheses [5,6] about the origin of such emissions, i.e., the so-called “imperfection theory” and the “coherence theory”. The first one claims that there is always a finite probability that molecules randomly excited by metabolic events can decay with the emission of photons instead of radiation-less dissipation processes. The second hypothesis assigns the emission to a coherent electromagnetic field generated within and between the cells by some biochemical reactions in which, perhaps, oxygen atoms are involved. As pointed out earlier, there are several experimental evidences that such radiation is a carrier of information between biological systems, thereby playing a fundamental role for cell to cell communication [10,11]. For example, the radiation emitted by growing plants or organisms can increase the rate of cell division in similar organisms by as much as 30%. This interesting phenomenon is called mitogenetic effect [12,13]. Recent research papers have also used the properties of biophotons to shed light into the processes of signal transmission in the brain [14,15,16].

The experimental set-up to measure the biophoton emission of lentil seeds is based on photomultiplier techniques, and it is made of a dark chamber and a photomultiplier sensitive to the visible energy range. The detector works as a photon counter with an efficiency given by the quantum efficiency of the photomultiplier, and the data are recorded as the number of photons detected, within a well-defined and adjustable time window of measurement, as a function of time. The measurements are taken during periods varying from a few hours to several days, depending on the sample. The typical photon-counting data set is a time series where the number of counted photons is reported in the ordinate axis as a function of time measured starting from a zero which can be chosen at will, and typically is the moment in which the measurement starts after having closed the dark chamber. Details will be given in Section 2, Material and Methods.

While the approximate ranges of intensity and energy of the biophoton spectra are fairly well- known, the statistical properties of photon emission are still a challenging issue, generating controversial claims. These properties are normally investigated by measuring the distribution of the counts established by photomultipliers in a time window of 1 s. Although claims of non-trivial statistical properties of biophoton emission, like coherence or squeezed states of light, are reported in the literature from the initial works of Popp in the 1980s, it is not yet clear if these properties properly represent biophoton light. See the review of Cifra et al. [17] for a complete discussion of these topics.

### 1.2. Introduction to Complexity

In this paper we do not focus on the importance of biophotons for communication, but we put our attention on the study of time series experimentally obtained by the photon counting, aiming to discuss the nature of the information time series. This important problem has been addressed in the recent work of Duan and co-workers [18] using permutation entropy (PE). This technique of complexity evaluation has been illustrated in detail in the earlier paper by Band and Pompe [19]. The measure of complexity generated by this technique is the so-called Lyapunov coefficient and since the PE is especially robust against noise and other perturbations, this makes hard to get reliable complexity evaluations. In the complexity literature there exists a wide consensus on the importance of Kolmogorov complexity [20] and especially of the Kolmogorov-Sinai entropy [21]. The evaluation of the Kolmogorov complexity has been the subject of extended literature. Although a deep discussion of this literature is out of the scope of this paper, we quote the discussion presented in Ref. [22]. These authors illustrated two research directions aiming at evaluating Kolmogorov complexity, one called compression aiming, as PE does, at establishing the Lyapunov coefficient and one called diffusion, which is based in converting the original time series into a diffusion process. The Kolmogorov complexity is turned into a scaling η, that is expected to depart from the ordinary value η = 0.5. Diffusion allows us to establish complexity also in the case where the Lyapunov coefficient vanishes.

The technique of analysis used here is based on the diffusion approach to Kolmogorov complexity and is called diffusion entropy analysis (DEA) [23,24]. As far as the important issue of noise filtering is concerned, which is the important benefit of PE [18,19], we note that DEA adopted in this paper is the refined version of [25] and that, as discussed in Section 3, this refined version yields the significant benefit of filtering the influence of non-crucial events, i.e., the FBM events defined in Section 3, and also of the noise.

Universal agreement on the origin of complexity does not exist yet. We find especially attractive the hypothesis of complexity emerging from self-organization. In general, a complex system is formed by several interacting units generating a whole with specific properties such as nonlinearity, self-similarity, self-organization, just to quote some of them. The complexity can be thought as a delicate balance between order and randomness, and when one of the two prevails complexity turns into simplicity.

One of the key features of most complex systems is the presence of the so-called renewal events [26,27]. The happening of a renewal event resets the memory of the system and the times between two successive renewal events are uncorrelated and independent. The probability distribution densities (pdd) of the time distance τ between two consecutive renewal events are called waiting time pdd and are denoted by the symbol ψ(τ). These pdd functions have the long-time form:

(1) $$\psi \left(\tau \right)\propto \frac{1}{\tau^{\mu }},$$

where *μ* is the complexity index that can go from 1 to ∞. A classic example of renewal event is a sudden change of direction in the flight of a swarm of birds. When a crucial event occurs, the birds fly in different directions making the global velocity of the swarm vanish. However, the crucial events activate a fast nucleation process, making the swarm select a new flying direction with no correlation with the earlier choices of flying direction [28,29]. The changes of flying directions are renewal events and the corresponding pdd has the inverse power law index *μ* = 1.35 [29]. These abrupt changes of directions are a form of intermittence generated by criticality. Contoyiannis et al. [30] studied the criticality-induced intermittence of the Ising model which is in line with the theoretical arguments of Schuster [31] and emphasized the emergence of intermittence with 1 < *μ* < 3 as a generator of 1/f noise, namely a noise with the spectrum P(f) given by:

(2) $$P\left(f\right)\propto \frac{1}{f^{\beta }}$$

with β=3−μ for 2 < *μ* < 3 and β=0 for μ>3. If μ>3 these renewal events generate the ordinary condition of white noise. If μ<3 Equation (2) indicates a departure from the condition of ordinary statistical physics, thereby leading us to the definition of crucial events adopted in this paper. Crucial events are renewal events corresponding to the condition 1<μ<3. These types of events are not confined only to the swarms of birds but can be found in many biological and physiological processes like, for example, heartbeat [32] and neural dynamic [27].

We believe that crucial events can be realized spontaneously by the complex systems along the recent lines presented in the work of Refs. [33,34,35] advocating self-organized temporal criticality (SOTC). SOTC is a theory explaining how a system of interacting units may spontaneously generate temporal complexity. It is a form of self-organized criticality characterized by crucial events. Currently, the theoretical approach of SOTC is limited to μ<2, but we make the plausible conjecture that this form of spontaneous organization may be extended to the whole crucial regime of μ<3.

In this paper we study the experimental biophoton emission of germinating lentils by using the DEA for the entire duration of the measurement, namely approximately 72 h from the closure of the germination chamber, to highlight any differences during the different stages of germination, and check for the presence of crucial events and determine the value of the *μ* parameter. The outline of this paper is as follows. In Section 2 we describe the experiment conducted to reveal the biophoton emission with a brief analysis of the count statistics in terms of photocount distributions. In Section 3 we illustrate the theoretical foundations of the DEA method used in the statistical analysis. In Section 4 we report the results of the DEA analysis discussing in detail the properties of crucial events present in the biophoton emission. We devote the last section to draw some conclusions and to discuss the possible role of quantum coherence in the late phase of the germination process which may become an important goal for future investigation.

## 2. Material and Methods

**Figure 1:**
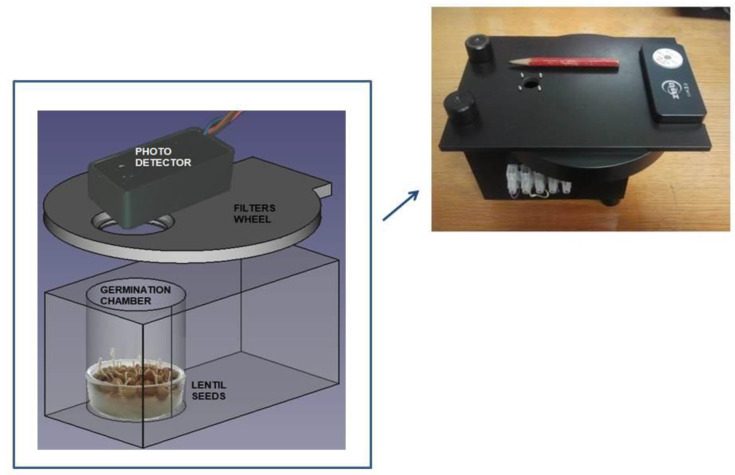
Technical design of the experimental setup used in our experiment. The photon-counting system consists of a Hamamatsu H12386-210 counting head. The germination chamber is built with black PVC to avoid any contamination of light from outside. At the top right a photo with the practical realization of our experimental set-up is shown.

Our experimental set up is composed of a home-made germination chamber, a photon counting system and a turning filters wheel. See Figure 1 for details. The photon counting device is a H12386-210 high-speed counting head (Hamamatsu Photonic Italia S.r.l, Arese (MI), Italy) powered at +5 Vcc. The phototube (with a bi alkali photocathode) is sensible in the wavelength range between 230 and 700 nm with a peak sensitivity at 400 nm. An ARDUINO (Ivrea, Italy) board, driven by a PC equipped with the Lab-VIEW program (National Instrument, Austin, TX, USA), is used for data acquisition and to control the experiment. The acquisition time window is fixed at 1 s at which the whole system shows a dark current of about 2 photon/sec at room temperature. Regarding the number of dark counts it should be specified that the figure reported on the Hamamatsu on-line catalog is an average value in the worst case [36]. Each photomultiplier is delivered with its own final test sheets which show the average value of the dark counts of that specific specimen. In our case, we have a value [37] of 1.7 counts/sec. A turning wheel holding a few long pass glass color filters is placed between the germinating seeds and the detector. The wheel has eight positions. Six are used for the color filters, one is empty and the last one is closed with a black cap.

Seeds are kept in a humid cotton bed placed in a Petri dish. In this experiment, the sample is composed by 75 lentil seeds. This number has been chosen to have a good signal-to-noise ratio. The lentils used in this experiment are normal seeds bought in a supermarket.

**Figure 2:**
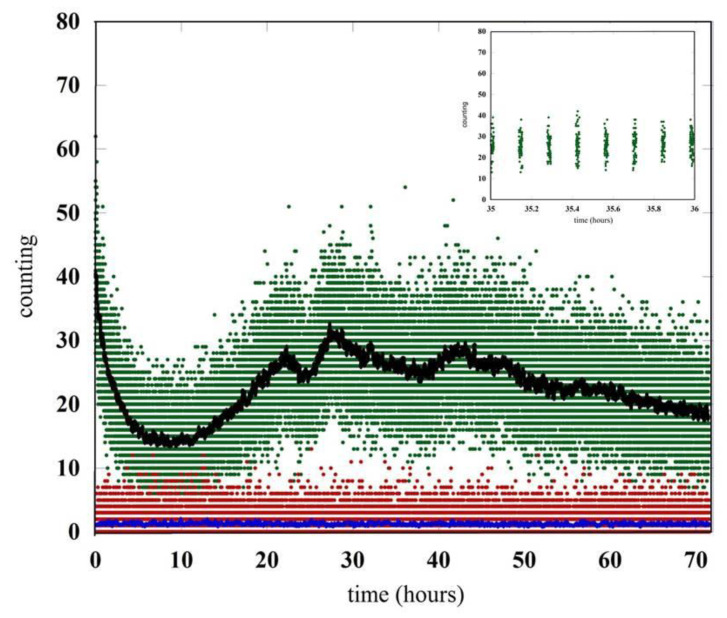
Comparison between the signal generated by the germinating seeds and the signal in the dark condition. The raw data are the green (seeds) and red (dark count) points. The black and blue curves are the raw data (count/sec) averaged over one minute. In the inset the emission of seeds for a period of one hour has been reported in detail.

Here, we only use and report the data coming from the empty and black cap positions. In this way, we have the total emission within the visible energy range and the dark counting of the phototube. The wheel stays in each position for one minute. This means that it returns to the same position after 420 s plus the time needed to switch from one position to the other, for a total of 443 s. For this reason the data have a bunch-type structure (see the inset of Figure 2). After one minute of measurement, there are 443 s of no data for a specific set of data.

Without any seeds or germination, there is a monotonic decrease in photon emission which arrives in a few hours to the value of the detector noise. This emission tail comes from the residual luminescence of the chamber materials, including the wet cotton bed and Petri dish, which is a consequence of the exposure to light of the equipment while setting up the experiment.

The comparison between the counting in the dark condition (red points) and the signal observed with the germinating seeds (green points) is reported in Figure 2. The data are related to an acquisition time of almost three days after the closing of the chamber. The emission of seeds for the period of time between hour 35 and hour 36 is shown in details in the inset of Figure 2. To clarify the behaviors of the different data sets, we also report the number of photons per second coming from the raw data averaged over one minute.

The residual luminescence of the whole equipment goes down in few hours up to the time when the lentils start germinating and the biophoton emission is strong enough to be detected. It is interesting to note how the biophotons emission changes during the time showing strong oscillations perhaps due to the different conditions of the seeds during the germination process. The initial behavior is dominated by the luminescence generated by the humid cotton bed. The germination-triggered biophoton emission emerges about ten hours after closing the chamber, and then it becomes dominant with a signal well above the detector noise for the entire duration of the experiment.

This type of temporal evolution of the light emission appears seemingly to be a general behavior of biophotons coming from seeds in the germination phase, for example Gallep and dos Santos [7] describe a very similar emission (see their Figure 1) concerning the germination of wheat seeds.

This behavior of the signal could be related to the spontaneous evolution to criticality described by Figure 3 of Ref. [33] where the initial mean field has the value of −1. The mean field then, as an effect of self-organization, moves through a fluctuating transient towards a condition of weaker fluctuations close to the maximal value of 1 signaling the achievement of criticality. The water here may activate a process of the same type, although criticality associated to germination, in the long-time regime, may be replaced by a different process characterized by the birth of the roots.

To conclude this paragraph, we report below a brief analysis of the emitted light in terms of counts distribution, that is the probability distribution function p(n,Δt) of finding n counts in the time interval Δt = 1 s, where 1 s is our acquisition time window. All experimental distributions were fitted with a normalized Poisson function $p\left(n\right)=\frac{\lambda^{n}}{n!}\cdot e^{-\lambda }$ varying the parameter λ, which is the average counting number 〈n〉 in our case.

**Figure 3:**
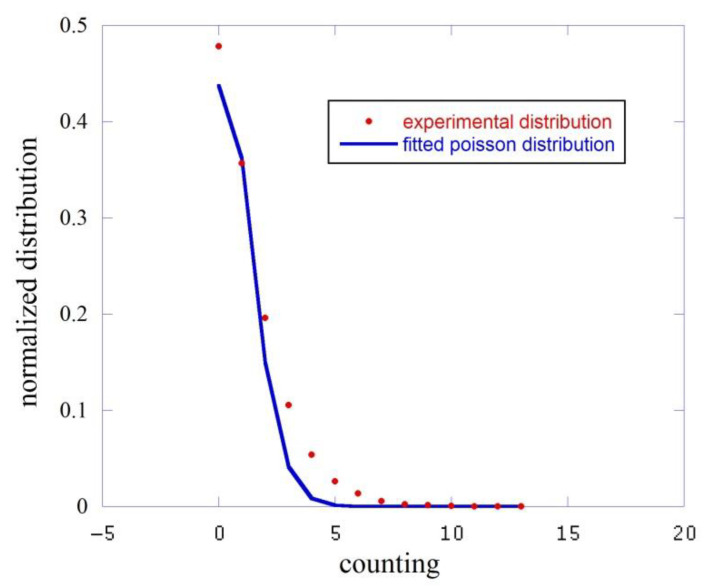
Comparison between the experimental counting distribution function (red squares) and the fit performed with a Poisson function (blue line).

In Figure 3 we report this analysis for the dark, i.e., the counts measured with the black cap. The red squares represent the experimental distribution while the blue line is the fitting function.

This fit gives an average counting value 〈n〉=0.827±0.03 count/sec to be compared to the experimental average 〈n〉=1.56 with a $\sigma^{2}=2.24$. We essentially measured here the dark counts of the phototube.

**Figure 4:**
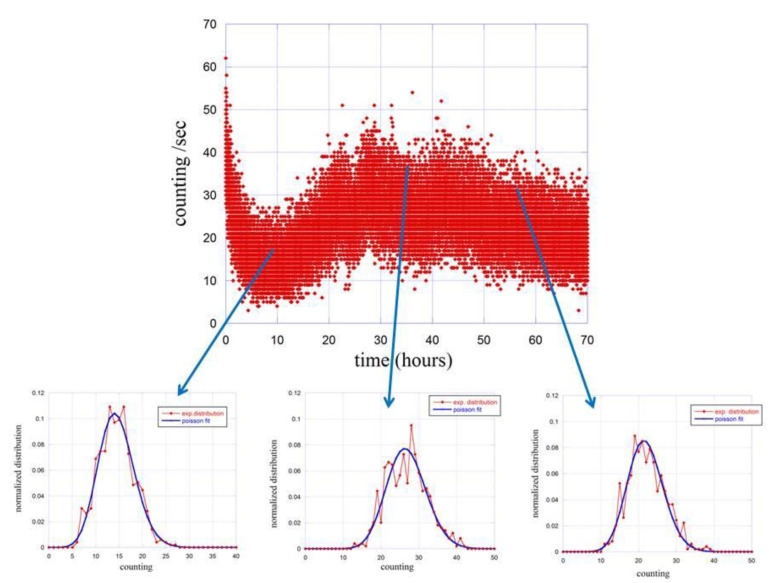
Time evolution of the counting distribution function p(n,Δt) (red dots) at different stage of the germinating process. Blue lines are fits done using Poisson functions.

We then proceeded with the same type of analysis to the emission in the presence of seeds. Due to the variation of the emission as function of the germination conditions, the analysis was carried out at different times using 1 h of emission to derive the probability distribution function p(n,Δt). The summary of the results is reported in Figure 4.

The probability distribution functions are calculated and fitted for three different times, namely at 10 h, 35 h and 55 h after the closure of the experimental apparatus. The obtained results are reported in Table 1.

Essentially, we observe a tendency to have a super-Poisson statistics, defined by the relation $\sigma^{2}>\langle n\rangle$, in all cases, which is a typical behavior either of a thermal emissions, where there are fluctuations in intensity on the time scales of the coherence time, or of a single source with a very short coherence time compared to the time interval of measurement. It should be noted that thermal light is characterized by the following relation between the variance and the mean value [38]:

(3) $$\sigma^{2}=\langle n\rangle +\frac{{\langle n\rangle }^{2}}{M},$$

where *M* is the number of the thermal modes. This number is generally large for a chaotic field so that the thermal field distribution tends to the Poisson distribution. In our cases *M* ranges from about 4 for the dark emission to about a thousand for the emission with seeds. This means that photocount statistics cannot discriminate between coherent and thermal states when many modes are present, as in the case with seeds, according to the discussion of Ref. [17].

## 3. Theoretical Foundation

The method of diffusion entropy analysis was introduced in 2001 in ref. [23]. It is based on converting the experimental time series, like the emission we record with our experimental set-up, into a diffusional trajectory. It is called diffusion entropy analysis because it determines the complexity of the signal through the evaluation of the Shannon entropy associated to the diffusional trajectory.

To achieve that we divide the time axis into bins of size s, and we assign to the n-th bin the value ξ(n), which is the number of photons emitted in that small-time interval. Consequently, the signal analyze is a time series {ξ(n)}. However, for notation simplicity, we discuss this analysis interpreting it as a continuous-time signal ξ(t), where ξ(t)=n(t) and n(t) is the number of photons emitted at time t. As done in the pioneering work [39], we plan to detect the complexity of ξ(t) studying the diffusional trajectory:

(4) $$x\left(t\right)=\int_{0}^{t}\xi \left(t^{\prime }\right)dt^{\prime }+x\left(0\right),$$

making the assumption that the complexity of the signal ξ(t) may be revealed by the anomalous scaling of the diffusional trajectory x(t).

Note that the analysis method adopted here, through the moving windows hereby described, is equivalent to assume that the time origin is at the space origin. Thus, we assume x(0)=0 and we observe the time series $x^{2}\left(t\right)$ rather than x(t). In fact, under the assumption 〈ξ(t)〉=0, the statistical observation of x(t) would turn out to be independent of ξ(t), which is the complex signal to be studied. There is no generality breaking here because we may turn the observation of ξ(t) into that of ξ(t)−〈ξ(t)〉. Then we make the average over many realizations of $x^{2}\left(t\right)$, yielding:

(5) $$\langle x^{2}\left(t\right)\rangle =\int_{0}^{t}dt_{1}\int_{0}^{t}dt_{2}\langle \xi \left(t_{1}\right)\xi \left(t_{2}\right)\rangle .$$

Following the traditional approach of assuming that correlation functions are stationary, we took that the correlation function $\langle \xi \left(t_{1}\right)\xi (t_{2})\rangle$ depends only on $\left|t_{1}-t_{2}\right|$, and it is totally independent of the absolute values of $t_{1}$ and $t_{2}$, which are two generic instants in the time series. This leads us to define the normalized correlation function:

(6) $$\Phi_{\xi }\left(\tau \right)=\frac{\langle \xi \left(t_{1}\right)\xi \left(t_{2}\right)\rangle }{\langle \xi^{2}\rangle },$$

where $\tau =\left|t_{1}-t_{2}\right|$.

The stationary assumption and the definition of Equation (6) allow us to rewrite the Equation (5) as:

(7) $$\langle x^{2}\left(t\right)\rangle =2\langle \xi^{2}\rangle \int_{0}^{t}dt^{\prime }\int_{0}^{t^{\prime }}dt^{\prime \prime }\Phi_{\xi }\left(t^{\prime \prime }\right).$$

The double integral in Equation (5) is evaluated by splitting it into two integrals, one with *t*′ moving from 0 to *t* and *t*″ moving from 0 to *t*′. The other integral is done integrating *t*″ from 0 to *t* and *t*′ from 0 to *t*″. The stationarity condition of Equation (6) allows us to prove that these two integrals are identical, leading through a simple change of integration variables to Equation (7).

We are now in the position to relate the complexity of ξ(t) to the anomalous scaling of the diffusion trajectory x(t). Using the Hurst [40] and the Fractional Brownian Motion [41] (FBM) notations, we indicate the scaling with the symbol *H*, namely we assume that $x\propto t^{H}$ and $\langle x^{2}\left(t\right)\rangle \propto t^{2H}$. Differentiating Equation (7) twice with respect to time, we get:

(8) $$\Phi_{\xi }\left(\tau \right)\propto 2H\left(2H-1\right)t^{2H-2},$$

This means that the deviation of *H* from the ordinary value *H* = 0.5 is due to the stationary correlation function $\Phi_{\xi }\left(t\right)$ which, in the long-time limit, has the structure:

(9) $$\Phi_{\xi }\left(\tau \right)\propto \pm \frac{1}{t^{\delta }},$$

with δ=2−2H. When *H* > 0.5 the parameter δ ranges from 0 to 1, generating a positive tail, while for *H* < 0.5 δ goes from 1 to 2, generating a negative tail [42].

It is interesting to note that a stationary correlation function with the same properties as of Equation (6) is obtained by assuming that the signal ξ(t) is a combination of harmonic oscillations cos(ωt) according to:

(10) $$\Phi_{\xi }\left(\tau \right)=\int^{\;}d\omega \rho \left(\omega \right)\cos\left(\omega t\right),$$

where $\rho \left(\omega \right)\propto \omega^{\delta -1}$, with the condition δ > 1 being defined as super-ohmic and the condition δ < 1 as sub-ohmic [43].

Although the conversion of the time series ξ(t) into a diffusion trajectory leads naturally to relate the complexity of ξ(t) to the Hurst coefficient H≠0.5, there exists another source of anomalous behavior of the diffusion trajectories that is described in detail, for instance, in Ref. [44]. There exist fluctuations called crucial events that cannot be described through stationary correlation functions. We remind here that the time distance between two consecutive crucial events is described by a waiting time distribution density ψ(τ) with the important time asymptotic property $\psi \left(\tau \right)\propto \frac{1}{\tau^{\mu }}$, and *μ* ranging from 1 to ∞. We can convert a sequence of crucial events into a diffusion trajectory by activating a random walker that in the correspondence of a crucial event makes a jump ahead of a constant fixed value, for instance 1. To distinguish the anomalous scaling generated by crucial events from other types of anomalous scaling, we denote it with the symbol η rather than *H*. It is shown [44] that:

(11) η=μ−1,

for 1<μ<2,

(12) $$\eta =\frac{1}{\mu -1},$$

for 2<μ<3, and

(13) $$\eta =\frac{1}{2},$$

for μ>3.

It is important to stress that the fluctuations generated by crucial events are stationary only for μ>3. If complexity is generated by crucial events, the region μ>3 is interpreted as corresponding to the manifestation of ordinary equilibrium statistical physics, and the region μ<3 as the land of non-stationary behavior, either temporary, 2<μ<3 or permanent for 1<μ<2.

The scaling evaluation implies that we observe many equivalent but distinct realizations of the same diffusional process. For the analysis of data generated by the emission of bio-photons we have at our disposal only one time series. We generate the diffusional trajectory x(t) according to the earlier prescription and, in order to make the statistical analysis, we convert this diffusional trajectory into many realizations such as to make it possible to do an ensemble average. These realizations are performed through a window of size l that we move along the trajectory x(t). Note that l cannot go to infinity because we work with a time series of length L. The largest value of l is M−t. For a window of length l ranging from t to t+l, we consider x(t) as the initial position of the random walker and we conclude that the random walker in time l moves from the origin to a value X=x(t+l)−x(t). Changing t we generate another realization X of the same length. Having L very large we have created a realizations number so big as to be able to evaluate the probability distribution density p(X,l) with a good accuracy, large enough to fit the scaling coefficient η, assuming the scaling condition:

(14) $$p\left(X,l\right)=\frac{1}{l^{\eta }}F\left(\frac{X}{l^{\eta }}\right),$$

To find η we evaluate first the Shannon entropy of the distribution density p(X,l) given by:

(15) $$S\left(l\right)=-\int_{-\infty }^{\infty }dx\;p\left(X,l\right)\ln\left[p\left(X,l\right)\right],$$

and later the scaling coefficient η plugging Equation (14) into Equation (15) obtaining with an easy algebraic calculation, that

(16) S(l)=A+η ln(l),

where *A* is a constant of no interest for our purposes. Thus, DEA allows us to measure the scaling coefficient η which is the slope of the straight line in the linear-log representation of S(l). In the case of ordinary Brownian diffusion η=0.5 and F(y) has a Gaussian form, while departure from this value indicates the presence of some complexity in the original time series. Of course, if the FBM condition applies η=H.

We move from the experimental data and generate the trajectory of Equation (4) which is converted into many realizations of size l with the moving window method. This method allows us to find the anomalous scaling, but does not allow to establish if it is due to a stationary or non-stationary correlation function.

To achieve that, we use DEA with the stripes prescription as done in Ref. [25]. We divide the ordinate of the ξ(t) fluctuations into many equal size bins and we detect the times at which the signal ξ(t) moves from one bin to one of the two neighboring bins. This allows us to define the occurrence of events, namely the crossing from one to the other bins. These events may be crucial or not. As explained in Ref. [25], the FBM fluctuations, if they exist, generate a scaling factor η=0.5 and the asymptotic S(l) depends only on the crucial events, if μ>1.5.

Notice that the choice of the size of the stripes is done with the criterion that the optimal size is between the extremely small and the very large. In between there exists a region where the scaling detected by DEA does not change with changing the stripe size. This is the size we adopted to determine the correct scaling.

In this paper we use DEA without and with stripes to establish if the anomalous scaling detected in our experimental data is due to FBM or to crucial events [25]. If H<η the two methods yield the same results for what concern the value of the scaling coefficient η, allowing us to state that the anomalous diffusion is due to crucial events. On the contrary, if H>η, the adoption of stripes filters the anomalous contribution generated by non-crucial events, including the FBM contribution. DEA without stripes yields H and, if η=0.5, we conclude that μ>3, implying that there are no crucial events. In this case, DEA without stripes yields a scaling coefficient larger than the one detected using stripes, this means that the complexity of the process is due to FBM.

The updated version of DEA used here is designed to determine if the signal complexity is generated by either non-ergodic crucial events with a non-stationary correlation function or by the infinite memory of a stationary but non-integrable correlation function or by a mixture of both processes. We have analyzed the dark count signal without germinating seeds as well as the photon emission during the germination process. We have found that dark count yields the ordinary scaling, thereby showing that no complexity of either kind may occur in the absence of any seed in the chamber. In the presence of seeds in the chamber anomalous scaling emerges. We show that in the first phase of germination the value of *μ* is less than 3, a value which increases with the progress of the germination process. In other words, the non-ergodic component tends to vanish with the time and complexity becomes dominated by the stationary infinite memory. We find here that the complexity is a mixture of the dynamics of crucial events and infinite memory that we interpret as a form of quantum coherence, which becomes predominant in the late phase of germination.

### Definition of μ

In the case when there are no crucial events and only FBM pseudo-events are detected, the connection between *μ* and the scaling η is given by:

η = (4 − μ)/2,(17)

In fact, the DEA without stripes does not make a distinction between crucial events and FBM. Correlation functions generated by crucial events with 2 < μ < 3 becomes stationary in the long-time limit, thereby corresponding to stationary correlation functions with the inverse power law index μ − 2 [40]. The inverse power law index of the correlation function of Equation (9), δ becomes:

δ = μ − 2,(18)

Thus, the scaling η is connected to δ by:

δ = 2 − 2 η,(19)

yielding Equation (17). Of course, the adoption of DEA with stripes would lead to a different scaling and the scaling detected using the DEA without stripes should be indicated by the symbol H.

It is important to notice that Equations (17) and (12) are appropriate for DEA without stripes and DEA with stripes, respectively. They produce the same result only for μ = 3 and μ = 2. However, the scaling for μ = 2.5 would be η = 0.67 for Equation (12) and η = 0.75 for Equation (17). We believe that the second result would provide an accurate information on H, while η = 0.67 would be an accurate information on the occurrence of crucial events with μ = 2.5.

## 4. Results

In this section we report the results obtained using DEA with and without stripes. The main items we want to discuss can be summarized in three questions: (a) Does μ changes with time through the germination process? (b) What are the values of *μ* at different stages of germination and how these compare with the values obtained in the analysis of some physiological processes, like in heartbeats [32] and brain dynamics studied by EEG recording [45,46]? (c) Does the analysis reveal a significant statistical difference between the dark counts and the signal with the seeds so as to make a plausible the conjecture that some type of complexity emerges during the germination process?

**Figure 5:**
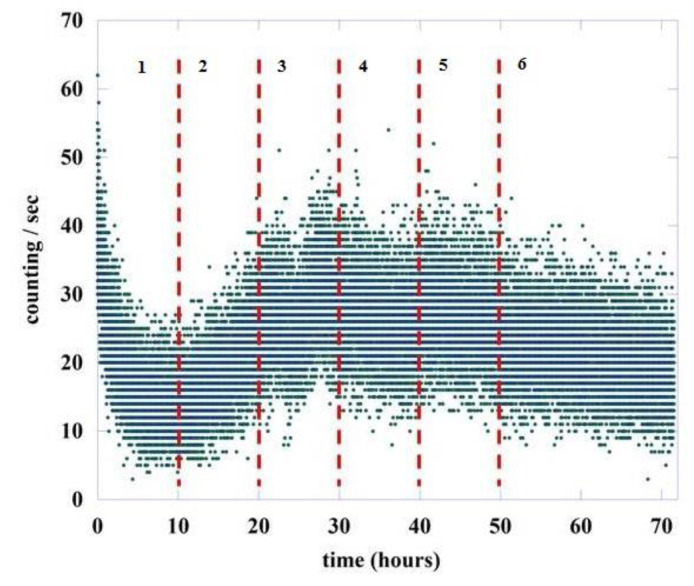
Number of photons emitted during the germination of lentil seeds (green points). The red dashed lines represent the six different regions used for the DEA analysis.

To understand how and if the value of μ changes during the germination process, we divided the total acquisition time of about 72 h into six regions, the first five having a length of 10 h while the last one is greater, being equal to 22 h. This is because experimentally a large variability is observed in the intensity of emission in the initial phases of the germination process, a variability that decreases in the final part of the recording. Figure 5 shows the six regions chosen for the analysis, separated by vertical red dashed lines.

**Figure 6:**
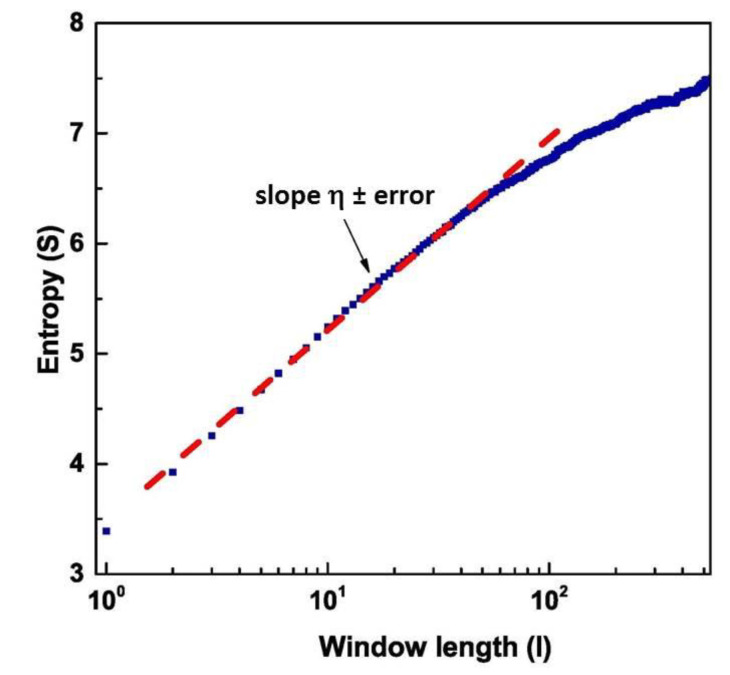
Graphical representation of the Diffusion Entropy Analysis method (blue curve). The red dashed line is the fit of the intermediate region.

Applying DEA to these different regions, we found the scaling factor η, according to Equation (16), and the corresponding μ using Equation (12). The scaling factor η is evaluated by fitting the slope of S(l) in an intermediate region between the short and large l values, using the concept of intermediate asymptotics [47]. The region of short values of l is a region of transition to complexity, while the region of large l values is either a region of transition to ordinary statistics or a region where the scarcity of events makes the evaluation of complexity inaccurate [33,34,35]. As an example, we have shown in Figure 6 the value of S(l) as a function of the length l of the window used for DEA, and the fit of the intermediate region to obtain the slope of the Shannon Entropy S(l). The data refers to the temporal region #1

This procedure has been done without and with stripes for all the six regions indicated in Figure 5 and for the dark counts, these last are not subdivided into six regions because they are practically constant throughout the measurement time, see the red points in Figure 2.

The obtained results are reported in Table 2. There are errors in the scaling factors determination that comes from the inaccuracy in determining the intermediate. These errors are of the order of 10^−4^ for the whole set of data and are not reported in the table for clarity.

We see from Table 2 that the analysis with no stripes yields a scaling significantly larger than the scaling obtained by DEA supplemented with stripes. Furthermore, while the scaling factor remains practically constant in all the six different regions when obtained by using the DEA without stripes, there is a significant time dependence for the scaling factor obtained by the DEA with stripes. This indicates that crucial events exist in the first three temporal regions, although the real value of μ, associated with this type of complexity, is closer to μ=3, the border with the region of ordinary statistical physics, than in other neuro-physiological processes in the human being [27,32,45,46].

For the last three temporal regions we cannot rule out the possibility that no crucial events exist and that the anomalous scaling, with a significant departure from the ordinary scaling η=0.5 is due to FBM. The authors of Ref. [48] with the help of Ref. [43] demonstrated that the dynamical origin of FBM is a generalized Langevin equation that is derived using the formalism of quantum mechanics [49], thereby suggesting that FBM may be a manifestation of quantum coherence. This answers to questions (a) and (b). The resulting values of μ do significantly change with time and remain in the region of μ significantly smaller than 3 only in the first three temporal regions.

As far as question (c) is concerned, we address this issue using again DEA without stripes and with stripes. The results obtained with stripes lead us to conclude that no temporal complexity exists in the dark counts time series. There is, however, a coherent contribution that is much weaker than the coherent contribution emerging in the long-time region of the germination process. It does not conflict with the main conclusion of this paper that a significant deviation from ordinary scaling either coherent or temporally complex is due to the germination process. We limit ourselves to notice that the dark state is just the electronic noise. The coherence of the dark state is something inherent to the instrument we are using that was never analyzed, to the best of our knowledge, with the DEA method thereby making it difficult to propose a plausible explanation which is out of scope of this paper.

## 5. Discussion and Conclusions

### 5.1. Results of This Paper

In the recent past the DEA method has been applied to a wide variety of problems, from the study of amino acid sequences in DNA [50], to Mozart’s music [51,52] up to the progression of autonomic neuropathy [53] looking for the heartbeat signal. Here, for the first time, this approach is applied to the biophotons emitted from germinating seeds.

The results of Table 2 show that in the first three germination regions a mixture of two conditions of anomalous diffusion is realized with a clear presence of crucial events enlighten by the fact that the analysis with the stripes gives values of *μ* substantially different from 3 in these first germination regions. On the other side, in the last three germination regions the analysis done with and without stripes suggests that the departure from the condition of random diffusion is due to FBM [54]. This seems to agree with the observation done by authors of a recent work on the auto-luminescence of germinating mango beans [55]. This conclusion would require further research investigation since the observation done by the authors of Ref. [55] is obtained comparing the experimental results to numerical calculations obtained running the FBM algorithm [41] for the observation of the process in the short time-scale while our theoretical analysis is done [42] focusing on the long-time limit.

We note that the values of *μ* obtained from the adoption of DEA without stripes cannot be trusted because are based on Equation (12), which would be correct in the absence of non-crucial events of FBM nature. We may use the values of *μ* obtained adopting DEA with stripes, using Equation (17). However, this has the effect of yielding a value for the scaling significantly larger than that achieved by the use the DEA with no stripes. This is a consequence again of the fact that the germination process generates both crucial events and non-crucial events of FBM type.

It is also important to notice that the authors of Ref. [53], who analyzed the heartbeats of patients under the influence of autonomic neuropathy, found that the increasing severity of this disease has the effect of making *μ* move from the healthy condition close to *μ* = 2 to the border with ordinary statistical physics, *μ* = 3, while the spectrum keeps the character of 1/f noise. It is so because the physiological system moves from a complexity condition generated by crucial events to a complexity condition characterized by the FBM infinite memory. In this paper we find equivalent results as made evident by Table 2 showing the DEA without stripes yields a scaling η that remains in the anomalous region 0.7 ÷ 0.8, which corresponds to 1/f noise. The real 1f noise has spectrum $P\left(f\right)\propto \frac{1}{f^{\beta }}\;$with β=3−μ when anomalous diffusion is generated by crucial events and β=3−2H when [54] the anomalous diffusion is only due to FBM. We note the deviation of the scaling *η* from the ordinary value 0.5 is always markedly larger if the scaling is evaluated without stripe, namely, when FBM is not filtered out yielding, for example, in region #5 the value *β* = 1.61.

The transition from regions with crucial events to regions where FBM dominates suggests the influence of quantum coherence [48]. Therefore, we are forced to adopt a physical interpretation of the transport of information that cannot rest only on the role of crucial events. Nevertheless, we think that our paper supports an important contribution to the revolution occurring in biology [56,57,58,59,60]. This is so because, to the best of our knowledge, in the current literature no research work has revealed the existence of crucial events in the germinating process. This paper shows that they exist at least in the first phase of the germination process. Although many authors emphasize the importance of quantum coherence for cognition, including authors believing that biophotons favor cell-to-cell communication, there is no prescription on how to measure and determine the intensity of quantum coherence. We believe that the results of this paper will lead to future research work where the role of quantum coherence can be established with more precision, by evaluating in detail the spectrum P(f) and deepening the quantum coherence properties discussed in Ref. [48].

In the literature about bio-photons there are indications that cell to cell communication through light is a plausible communication mechanism, however, there are obstacles to proving it due to the unfavorable signal-to-noise ratio of the signal detection by cells [11]. The authors of Ref. [11] discuss the limitations of the famous theory of stochastic resonance. We want to stress that the complexity matching theory of [61] is a much more advanced approach making it possible to establish communication through one single realization rather than an average over an ensemble of realizations, compatible with the joint action of coherence and crucial events. This interpretation may lead to an agreement with that adopted, for instance by Fels [12] where the belief is that the transfer of information and the intelligence itself are based on quantum coherence [6,62]. In our perspective, however, the origin of cognition is a process of self-organization [33,34] of the components of the seeds corresponding to water-induced germination. This process of self-organization generates both crucial events and quantum coherence.

Our analysis clearly shows the presence of crucial events, at least in the early stages of the germination process, and the presence of a transition to a stage where the scaling remains significantly anomalous, but the main origin of this anomaly is due to FBM which, according to Ref. [48], may be a sign of quantum coherence. The advocates of quantum mechanics to explain the origin of cognition may use these results to support their view. We limit ourselves to notice that other conjectures may be invoked, for instance the influence of stress on the biophoton emission [63,64]. In this case, the stress may be related to the fact that the seedlings begin to grow in an environment completely devoid of light.

It is interesting to note that while in human beings the presence of crucial events is a necessary condition to make different organs "talk" to each other, for example the heart and brain, and that a transition to a situation in which complexity is mainly due to FBM is a transition from a health condition to a pathological degeneration, this is not true for plants. But this may not be surprising, plants do not have well-defined organs, only at the beginning of the germination process there is a differentiation process that may require the presence of crucial events. In other words, we cannot rule out that the kind of criticality involved by the germination process requires a form of phase transition that is not yet known. It has to be stressed that Mancuso [65] and Mancuso and Viola [66] use the concept of swarm intelligence with reference to the non-hierarchical root network. Thus, it may be beneficial to supplement their observations noting that the initial region of germination may have to do with the birth of this surprising root intelligence.

### 5.2. Suggestions for Future Research Work

We notice an increasing interest in the literature for the role that biophotons may have in biology, physiology, for the dynamics of the brain and for the challenging issue of emergence of cognition [14,67,68,69]. The authors of these interesting papers adopt different views, ranging from the role of biophotons as essential to establish links between different organs of the human body to the interaction between different organs as being itself a quantum mechanical property [15]. This paper affords a technique of statistical analysis leading to the conclusion that the FBM infinite memory advocated by the authors of Ref. [14] is indeed an important ingredient of the physiological mechanism of information coding, transmission, and processing in the nervous system. However, we do not rule out the connection with self-organized criticality that may be the source of the joint action of the infinite FBM memory and crucial events [53]. We shall explore this important issue by comparing the transition to FBM induced by autonomic neuropathy disease in human beings [53] to the hypotheses of stress signaled by biophoton emission [63,64]. We hope that the present paper may contribute to shedding further light on the role played by biophotons on these processes.

## Figures and Tables

**Table 1:** Summary of the numerical results obtained by the counting distribution analysis.

	Exp. <*n*>	Exp. σ^2^	Poisson <*n*>
dark	1.56	2.24	0.827 ± 0.03
1	14.40	14.60	14.51 ± 0.10
2	26.83	29.12	26.80 ± 0.21
3	22.21	24.84	21.90 ± 0.13

**Table 2:** The scaling factors obtained by DEA without and with stripes for the six different regions of the time series with seeds and for the whole region of the dark counts time series.

	Without Stripes		With Stripes	
	η	μ	η	μ
dark counts	0.575	2.739	0.508	2.968
region #1	0.773	2.293	0.596	2.677
region #2	0.796	2.254	0.558	2.792
region #3	0.736	2.358	0.526	2.901
region #4	0.737	2.355	0.496	3.016
region #5	0.694	2.440	0.509	2.964
region #6	0.725	2.377	0.504	2.984

## Data Availability

All relevant data are available from the authors upon request.

## References

[1] Gurwitsch A.G. (1923). Die Natur des spezifischen Erregers der Zellteilung. Arch. Entw. Mech. Org..

[2] Reiter T., Gabor D. (1928). Ultraviolette Strahlung und Zellteilung. Wiss. Verffentlichungen Aus Dem Siemens-Konzern.

[3] Colli L., Facchini U. (1954). Light Emission by Germinating Plants. Il Nuovo Cim..

[4] Colli L., Facchini U., Guidotti G., Dugnani Lonati R., Orsenigo M., Sommariva O. (1955). Further Measurements on the Bioluminescence of the Seedlings. Experientia.

[5] Popp F.A., Gu Q., Li K.H. (1994). Biophoton Emission: Experimental Background and Theoretical Approaches. Mod. Phys. Lett. B.

[6] Van Wijk R. (2014). Light in Shaping Life: Biophotons in Biology and Medicine.

[7] Gallep C.M., Dos Santos S.R. (2007). Photon-count during germination of wheat (Triticum aestivum) in waste water sediment solution correlated with seedling growth. Seed Sci. Technol..

[8] Musumeci F., Scordino A., Triglia A., Blandino G., Milazzo I. (1999). Intercellular communication during yeast cell growth. Europhys. Lett..

[9] Grasso F., Grillo C., Musumeci F., Triglia A., Rodolico G., Cammisuli F., Rinzivillo C., Fragati G., Santuccio A., Rodolico M. (1992). Photon emission from normal and tumor human tissue. Experientia.

[10] Mauburov S.N. (2011). Photonic Communications in Biological Systems. J. Samara State Tech. Univ. Ser. Phys. Math. Sci..

[11] Kucera O., Cifra M. (2013). Cell-to-cell signaling through light: Just a ghost of chance?. Cell Comm. Signal..

[12] Fels D. (2009). Cellular Communication through light. PLoS ONE.

[13] Beloussov L.V., Burlakov A.B., Louchinskaia N.N. (2003). Biophotonic Pattern of optical interaction between fish eggs and embryos. Indian J. Exp. Biol..

[14] Tang R., Dai J. (2014). Biophoton signal transmission and processing in the brain. J. Photochem. Photobiol. B Biol..

[15] Kumar S., Boone K., Tuszynski J., Barclay P., Simon C. (2016). Possible existence of optical communication channels in the brain. Sci. Rep..

[16] Zangari A., Micheli D., Galeazzi R., Tozzi A. (2018). Node of Ranvier as an Array of Bio-Nanoantennas for Infrared Communication in Nerve Tissue. Sci. Rep..

[17] Cifra M., Brouder C., Nerudova M., Kucera O. (2015). Biophotons, coherence and photocount statistics: A critical review. J. Lumin..

[18] Duan S., Wang F., Zhang Y. (2019). Research on the biophoton emission of wheat kernels based on permutation entropy. Opt. Int. J. Light Electron Opt..

[19] Bandt C., Pompe B. (2002). Permutation Entropy: A Natural Complexity Measure for Time Series. Phys. Rev. Lett..

[20] Falconi M., Loreto V., Vulpiani A. (2003). Kolmogorov Legacy about Entropy, Chaos and Complexity.

[21] Latora V., Baranger M. (1999). Kolomogorov-Sinai Entropy Rate versus Physical Entropy. Phys. Rev. Lett..

[22] Allegrini P., Benci V., Grigolini P., Hamilton P., Ignaccolo M., Menconi G., Palatella L., Raffaelli G., Scafetta N., Virgilio M. (2003). Compression and diffusion: A joint approach to detect complexity. Chaos Solitons Fractals.

[23] Scafetta N., Hamilton P., Grigolini P. (2001). The thermodynamics of social processes: The teen birth phenomenon. Fractals.

[24] Scafetta N., Grigolini P. (2002). Scaling detection in time series: Diffusion Entropy analysis. Phys. Rev. E.

[25] Culbreth G., West B.J., Grigolini P. (2019). Entropic Approach to the Detection of Crucial Events. Entropy.

[26] Grigolini P. (2015). Emergence of biological complexity: Criticality, renewal and memory. Chaos Solitons Fractals.

[27] Bohara G., West B.J., Grigolini P. (2018). Bridging Waves and Crucial Events in the Dynamic of the Brain. Front. Physiol..

[28] Attanasi A., Cavagna A., Del Castello L., Giardina I., Melillo S., Parisi L., Pohl O., Rossaro B., Shen E., Silvestri E. (2014). Finite-Size Scaling as a Way to Probe Near-Criticality in Natural Swarms. Phys. Rev. Lett..

[29] Vanni F., Luković M., Grigolini P. (2011). Criticality and Transmission of Information in a Swarm of Cooperative Units. Phys. Rev. Lett..

[30] Contoyiannis Y.F., Diakonos F.K., Malakis A. (2002). Intermittent Dynamics of Critical Fluctuations. Phys. Rev. Lett..

[31] Schuster H.G. (1988). Deterministic Chaos.

[32] Bohara G., Lambert D., West B.J., Grigolini P. (2017). Crucial events, randomness and multifractality in heartbeat. Phys. Rev. E.

[33] Mahmoodi K., West B.J., Grigolini P. (2017). Self-Organizing Complex Networks: Individual versus global rules. Front. Physiol..

[34] Mahmoodi K., West B.J., Grigolini P. (2018). Self-Organized Temporal Criticality: Bottom-up resilience versus top-down vulnerability. Complexity.

[35] Mahmoodi K., Grigolini P., West B.J. (2018). On social sensitivity to either zealot or independent minorities. Chaos Solitons Fractals.

[36] Photon Counting Head H12386-210 Datasheet. https://www.hamamatsu.com/eu/en/product/type/H12386-210/index.html.

[37] Test Sheet Hamamatsu for the Phototube H12386-210, Serial Number 30050260. https://www.hamamatsu.com/resources/pdf/etd/H12386_TPMO1073E.pdf.

[38] Fox M. (2006). Quantum Optics—An Introduction.

[39] Stanley H.E., Buldyrev S.V., Goldberger A.L., Goldberger Z.D., Havlin S., Mantegna R.N., Ossadnik S.M., Peng C.K., Simons M. (1994). Statistical mechanics in biology: How ubiquitous are long-range correlations?. Physica A.

[40] Hurst H.E. (1951). Long-term storage capacity of reservoirs. Trans. Am. Soc. Civ. Eng..

[41] Mandelbrot B.B., Walls J.R. (1968). Noah, Joseph and operational hydrology. Water Resourc. Res..

[42] Cakir R.A.Ş.İ.T., Grigolini P., Krokhin A.A. (2006). Dynamical origin of memory and renewal. Phys. Rev. E.

[43] Weiss U. (1992). Quantum Dissipative Systems.

[44] Grigolini P., Palatella L., Raffaelli G. (2001). Anomalous Diffusion: An Efficient Way to Detect Memory in Time Series. Fractals.

[45] Paradisi P., Allegrini P., Gemignani A., Laurino M., Menicucci D., Piarulli A. (2013). Scaling and intermittency of brain events as a manifestation of consciousness. AIP Conference Proceedings.

[46] Allegrini P., Menicucci D., Bedini R., Fronzoni L., Gemignani A., Grigolini P., West B.J., Paradisi P. (2009). Spontaneous brain activity as a source of ideal 1/f noise. Phys. Rev. E.

[47] Barenblatt G.I. (1996). Scaling, Self-Similarity and Intermediate Asymptotics.

[48] Culbreth G., West B.J., Grigolini P. (2021). Caputo Fractional Derivative versus Quantum Coherence. Entropy.

[49] Mori H. (1965). Transport, Collective Motion and Brownian Motion. Prog. Theor. Phyiscs.

[50] Scafetta N., Latora V., Grigolini P. (2002). Levy scaling: The diffusion entropy analysis applied to DNA sequences. Phys. Rev. E.

[51] Vanni F., Grigolini P. (2017). Music as a mirror of mind. Esthétique de la Complexité.

[52] Pease A., Mahmoodi K., West B.J. (2018). Complexity measures of music. Chaos Solitons Fractals.

[53] Jelinek H.F., Tuladhar R., Culbreth G., Bohara G., Cornforth D., West B.J., Grigolini P. (2020). Diffusion Entropy versus Multiscale and Renyi Entropy to detect progression of Autonomic Neuropathy. Front. Physiol..

[54] Lukovic M., Grigolini P. (2008). Power spectra for both interrupted and perennial aging process. J. Chem. Phys..

[55] Dlask M., Kukal J., Poplová M., Sovka P., Cifra M. (2019). Short–time fractal analysis of biological autoluminescence. PLoS ONE.

[56] Miller W.B. (2018). Biological information systems: Evolution as cognition-based information management. Prog. Biophys. Mol. Biol..

[57] Eigen M. (2013). From Strange Simplicity to Complex Familiarity: A Treatise on Matter, Information, Life and Thought.

[58] Ford B.J. (2009). On Intelligence in Cells: The Case for Whole Cell Biology. Interdiscip. Sci. Rev..

[59] Dodig-Crnkovic G., Beckmann A., Csuhaj-Varj E., Meer K. (2014). Modeling Life as Cognitive Info-Computation. Language, Life, Limits.

[60] Maturana H., Varela F. (1980). Autopoiesis and Cognition: The Realization of the Living.

[61] Mahmoodi K., West B.J., Grigolini P. (2019). Complexity Matching and Requisite Variety. arXiv.

[62] Popp F.A. (2008). Consciousness as Evolutionary Process Based on Coherent States. NeuroQuantology.

[63] Piao D. (2020). On the stress-induced photon emission from organism: II, how will the stress-transfer kinetics affect the photo-genesis?. SN Appl. Sci..

[64] Piao D. (2020). On the stress-induced photon emission from organism: I, will the scattering-limited delay affect the temporal course?. SN Appl. Sci..

[65] Mancuso S. (2018). The Revolutionary Genius of Plants: A New Understanding of Plant Intelligence and Behaviour.

[66] Mancuso S., Viola A. (2015). Brilliant Green: The Surprising History and Science of Plant Intelligence.

[67] Van Wijk R., Van Wijk E., Pang J., Yang M., Yan Y., Han J. (2020). Integrating Ultra-Weak Photon Emission Analysis in Mitochondrial Research. Front. Physiol..

[68] Prasad A., Gouripeddi P., Devireddy H.R.N., Ovsii A., Rachakonda D.P., Wijk R.V., Pospíšil P. (2020). Spectral Distribution of Ultra-Weak Photon Emission as a Response to Wounding in Plants: An in Vivo Study. Biology.

[69] Dal Lin C., Falanga M., De Lauro E., De Martino S., Vitiello G. (2020). Biochemical and biophysical mechanisms underlying the heart and the brain dialog. Biophysics.

